# The Association With Two Different Arbuscular Mycorrhizal Fungi Differently Affects Water Stress Tolerance in Tomato

**DOI:** 10.3389/fpls.2018.01480

**Published:** 2018-10-09

**Authors:** Veronica Volpe, Walter Chitarra, Pasquale Cascone, Maria Grazia Volpe, Paola Bartolini, Gloriano Moneti, Giuseppe Pieraccini, Claudia Di Serio, Biancaelena Maserti, Emilio Guerrieri, Raffaella Balestrini

**Affiliations:** ^1^Department of Life Sciences and Systems Biology, University of Turin, Turin, Italy; ^2^Council for Agricultural Research and Economics, Centre of Viticulture and Enology Research, Conegliano, Italy; ^3^National Research Council, Institute for Sustainable Plant Protection, Turin-Florence-Portici (NA) Units, Portici, Italy; ^4^CNR, Institute of Food Sciences, Avellino, Italy; ^5^Department of Health Sciences, University of Florence, Florence, Italy; ^6^Geriatric Intensive Care Unit, Experimental and Clinical Medicine Department, University of Florence, AOU Careggi, Florence, Italy

**Keywords:** aphid, arbuscular mycorrhizal symbiosis, phosphate transporter, plant tolerance, *Solanum lycopersicum*, volatile organic compound, water deficit

## Abstract

Arbuscular mycorrhizal (AM) fungi are very widespread, forming symbiotic associations with ∼80% of land plant species, including almost all crop plants. These fungi are considered of great interest for their use as biofertilizer in low-input and organic agriculture. In addition to an improvement in plant nutrition, AM fungi have been reported to enhance plant tolerance to important abiotic and biotic environmental conditions, especially to a reduced availability of resources. These features, to be exploited and applied in the field, require a thorough identification of mechanisms involved in nutrient transfer, metabolic pathways induced by single and multiple stresses, physiological and eco-physiological mechanisms resulting in improved tolerance. However, cooperation between host plants and AM fungi is often related to the specificity of symbiotic partners, the environmental conditions and the availability of resources. In this study, the impact of two AM fungal species (*Funneliformis mosseae* and *Rhizophagus intraradices*) on the water stress tolerance of a commercial tomato cultivar (San Marzano nano) has been evaluated in pots. Biometric and eco-physiological parameters have been recorded and gene expression analyses in tomato roots have been focused on plant and fungal genes involved in inorganic phosphate (Pi) uptake and transport. *R. intraradices*, which resulted to be more efficient than *F. mosseae* to improve physiological performances, was selected to assess the role of AM symbiosis on tomato plants subjected to combined stresses (moderate water stress and aphid infestation) in controlled conditions. A positive effect on the tomato indirect defense toward aphids in terms of enhanced attraction of their natural enemies was observed, in agreement with the characterization of volatile organic compound (VOC) released. In conclusion, our results offer new insights for understanding the molecular and physiological mechanisms involved in the tolerance toward water deficit as mediated by a specific AM fungus. Moreover, they open new perspectives for the exploitation of AM symbiosis to enhance crop tolerance to abiotic and biotic stresses in a scenario of global change.

## Introduction

In the last decade, climatic change heavily influenced environmental conditions and a negative impact on plant development and productivity has been recorded. Plant responses to the environmental stresses include morphological, physiological, cellular and molecular changes (reviewed in Gray and Brady, 2016). One of the major abiotic threats on agriculture productivity is the progressive diffusion of water deficit in different areas of world (Dai, 2011), which induces a reduction in plant growth and development. Water deficit causes stomatal closure with a consequent decrease of CO_2_ adsorption followed by a reduction in photosynthetic activity and carbon partitioning (Osakabe et al., 2014; Chitarra et al., 2016). Additionally, drought has a negative impact on nutrient supply, leading to a decrease in phosphate availability (Sardans and Peñuelas, 2004). Root growth and development are important traits for plant survival in water stressed soil. A deep and extensive root system enables plants to access moisture in water-absorbed zones of soils, and is thus considered as a vital strategy for drought adaptation (Zou et al., 2017). In many crops (e.g., rice, wheat, and maize) an alteration of root system architecture has been observed under water deficit conditions, with a consistent increase in the elongation of primary roots, in the total root biomass and in the production of lateral roots, along with a change in the root growth angle and a reduction of root diameter (Comas et al., 2013). Root responses to drought stress depend on the induction of abscisic acid (ABA) pathway. This hormone is involved in different root cellular responses such as the activation of enzymes involved in cell wall remodeling (Wu et al., 1994), the regulation of cell type-specific development (Enstone et al., 2002) and the increase of lateral roots (De Smet et al., 2003). However, in agro-ecosystems, in addition to water limitation, crops have often to face the concurrent presence of other abiotic and biotic stresses, e.g., insect pests and pathogens (Bai et al., 2018). Hence, plants have to modulate a multifaceted defense by activating a number of molecular, biochemical and morphological changes, which can differ from the responses to a single stress factor (Atkinson and Urwin, 2012). Different plant defense responses lead to the release of volatile organic compounds (VOC) blends having different composition in term of presence or quantity of single compounds, depending on the stress features (Holopainen and Gershenzon, 2010). VOC released in response to pest attack can have direct and indirect defensive effect on insect performance. For example, methyl salicylate and the *cis*-3-hexen-1-ol, released by tomato plants in response to aphid attack (Sasso et al., 2007), have negative effects on subsequent aphid fixation (Digilio et al., 2012). These same compounds are powerful attractant of aphid natural enemies (Sasso et al., 2007, 2009). Moreover, the response of different genotypes, i.e., commercial genotypes or traditional varieties/landraces adapted to local environmental conditions, to stresses may be different. In this scenario, there is a consolidated evidence of the positive role played by “biostimulants,” e.g., organic (humic acids and seaweed extracts) and inorganic (silicium) substances, and/or microorganisms, which can be applied to improve plant nutrition/growth and to protect against stresses (du Jardin, 2015). Among them, great interest has been developed in the last years to beneficial soil microorganisms, including arbuscular mycorrhizal (AM) fungi, with a role to improve plant nutrition and tolerance to several environmental stresses. AM symbiosis is one of the most ancient interactions between the roots of more than 80% of terrestrial plants, including several crops (Balestrini and Lumini, 2018). During this interaction, in addition to the improvement of plant nutritional status, AM fungi can promote plant performance and protection from various stresses, including water deficit (Lenoir et al., 2016; Balestrini et al., 2018). In addition to an improved root system capacity to absorb nutrients and water due to the presence of external fungal hyphae, it has been also demonstrated that AM colonization influences the architecture of the host root system, leading to a better adaptation of morphology in response to water stress (Gutjahr et al., 2009; Fusconi, 2014; Zou et al., 2017). Recently, Chitarra et al. (2016) showed that the AM symbiosis positively affects the tomato tolerance to severe water deficit and how the adaptive plant response is dependent on the AM fungi species involved, underlying the importance to identify the optimal genotype/microorganism(s) combination to maximize plant resilience. Considering the role of isoprenoids in resistance to stress and plant defense, Asensio et al. (2012) tested the interaction between tomato plants and a mixed AM fungal inoculum under a drought stress conditions or jasmonic acid (JA) application, demonstrating an impact of the AM colonization in the pathways involved in isoprenoid production mainly under the considered treatments (drought and JA).

However, although the effects of AM symbiosis on the interactions with aphids have been already reported (Babikova et al., 2013, 2014), scarce information is available on these interactions under an abiotic stress condition such as water stress.

In this work, a multidisciplinary approach, involving eco-physiological, morphometric, biochemical and molecular analyses, and targeted metabolomics, has been used. Particularly, the mechanisms and the species-specificity of AM symbiosis in plant tolerance to moderate (MS) and severe (SS) water stress have been evaluated in comparison with unstressed plants. For this purpose, we tested two AM fungi, *Funneliformis mosseae* and *Rhizophagus intraradices*, in their association with a cultivated tomato cultivar (i.e., San Marzano nano). In particular, root gene expression analyses were focused on plant and fungal genes involved in phosphate (Pi) uptake and transport because of the importance of phosphorous use efficiency (PUE) in plants grown under abiotic stress (reviewed in Wang et al., 2017). It has been recently reported that, Pi transporter genes are regulated by a drought stress, both in poplar (Zhang et al., 2016) and apple (Sun et al., 2017). Here, we aimed to assess whether this applies to tomato and mainly how different AM fungi alter the expression of these genes under moderate (MS) and severe (SS) water stress conditions. In addition, considering that in the field plants are usually subjected to multiple stresses, the AM fungus (*R. intraradices*), resulting more efficient in increasing water stress tolerance, was selected to assess its role on tomato resistance to a combination of abiotic (water deficit) and biotic (aphid attack) stresses.

## Materials and Methods

### Plant, Fungal Material, and Experimental Design

*Solanum lycopersicum cv*. “San Marzano nano” (i.e., dwarf) seeds were surface-sterilized in sodium hypochlorite 5% (NaOCl) for 20 min, washed five times in sterile water, and germinated on wet paper. Seedlings were then moved to pots (10 × 10 × 12) containing a mixture of quartz sand (50%), sterile pumice (20%), and an inoculum (30%) of either *Funneliformis mosseae* (formerly *Glomus mosseae*; BEG 12) or *Rhizophagus intraradices* (FR 121), both purchased from MycAgro (Bretenière, France^1^). Propagules of AM fungi (i.e., a mix of spores, mycelium and mycorrhizal root pieces) are minimum equal to 10 propagules/g. For non-colonized plants (Ctrl), sterile inoculum carrier only (i.e., mix of inert mineral predominantly made with zeolite) was used instead of the specific inoculum (30%). For each pot, a quantity of about 650 g of sand plus inoculum/carrier has been used. Plants were grown in controlled conditions, at 23/21 ± 1°C (day/night), 16/8 h light/dark photoperiod, and 65 ± 10% relative humidity. From transplanting to the beginning of the experiment (after about 6/7 weeks), all the plants were watered twice a week with tap water and, once a week, with a modified Long-Ashton nutrient solution (Hewitt, 1966) containing 300 μM [Pi]. Two independent experiments (Experiment 1 and 2) were performed to determine the role of AM symbiosis under water deficit.

### Experiment 1

This experiment was devoted to verify the impact of the two different AM fungi on the regulation of Pi transporter genes in roots and on the production of specific metabolites in leaves. Biometric and eco-physiological parameters have been also registered to verify the plant stressed status. Considered treatments were: (i) AM fungal colonization [non-colonized (Ctrl), *F. mosseae*-colonized (Fmos) and *R. intraradices*-colonized (Rin)] and (ii) water stress [none (NS), moderate (MS), and severe (SS)]. For each condition, 12 plants were maintained in a well-watered state (at container capacity, irrigated or unstressed, NS). The remaining 24 plants were subjected to two water stress levels. Irrigation was withheld about 6/7 weeks after fungal inoculation: moderate stress (MS) was achieved in about 2 weeks (leaf water potential of about -0.9 MPa), whilst 3 weeks were necessary to produce a severe water stress (SS) status (petiole water potential lower than -1.0 MPa). From the beginning of the water deficit, plants were moved (at least one time a week) inside the climatic chamber to avoid positional differences. Root colonization has been assessed at the beginning and at the end of the water stress imposition (not shown).

#### Biometric and Physiological Measurements

At the end of the water stress experiment (about 10 weeks after the beginning of the experiment), plants were harvested and plant height and shoot diameter were recorded. Parameters were taken as reported by Chitarra et al. (2016). Briefly, leaf water potential (Ψ_leaf_) was measured on one transpiring leaf per plant, using a Scholander-type pressure chamber (Soil Moisture Equipment Corp., Santa Barbara, CA, United States). Measurements of transpiration rate (E), stomatal conductance (g_s_), and net photosynthetic rate (A_N_) were performed on adult, non-senescing, leaves at the same physiological age (in the middle part of the plant, considering the third-fourth leaf form the shoot apex). Intrinsic water use efficiency (iWUE) was calculated as the ratio between A_N_ and g_s_. Measurements were taken with an infrared gas analyzer ADC-LCPro+ system (The Analytical Development Company Ltd., Hoddesdon, United Kingdom). During measurement, light intensity in the leaf chamber was set at 1200 μmol photons m^-2^ s^-1^, temperature was 25°C, and concentration of CO_2_ was maintained at 450–470 ppm. Measurements were taken between 10:00 and 13:00 h. The chlorophyll content index (CCI) was determined at the end of the experiment (16 or 20 days after treatment, DAT) using the portable chlorophyll meter SPAD 502 (CCM-200, Opti-Sciences, Inc., Hudson, NH, United States). Readings were collected from the second or third fully developed leaves from the top on five randomly selected tomato plants for each experimental condition.

#### Phosphorus Determination

Analytical grade reagents and Phosphorus (P) standard (1000 mg/L) were purchased from Sigma-Aldrich chemical. The digestion of leaf samples was performed according to Olowu et al. (2015) with some modifications. 0.10 g of each sample was weighed and 3 mL of a mixture of nitric acid: perchloric (1: 1; v: v) was added to each. The mixture was placed in a digester at 220°C for about 60 min. After 45 min, 500 μL of H_2_O_2_ (30%) were added in order to facilitate the oxidation reaction. After cooling the whole was transferred into a 10 mL volumetric flask and brought to volume with H_2_O. An Inductively Coupled Argon Plasma Optical Emission Spectrometers (ICP-OES iCAP 7000 Series Thermo Scientific), equipped with ASX-520 Autosampler (CETAC^TM^, Thermo Scientific) was used. To prepare the calibration curve with the Phosphorus concentrations equal to 0.5; 1; 2; 4 and 5 mg/L, the Multi-Element Test Solution ICAP 6000 with P concentration of 10 mg/L in 1% v/v HNO_3_ was used. The element content was calculated by using standard curves and the final concentrations of samples were expressed as g/kg dry weight of P. The emission line for the analysis by ICP OES was chosen according to previous interference studies. The line that exhibited low interference and high analytical signal and background ratios was selected. The emission line that was employed was 185.942 nm. The ICP-OES measurements were performed in triplicate, with *R*^2^ = 0.9994 and BEC (Background Equivalent Concentration) = 0.002 ppm.

#### RNA Extraction and RT-qPCR

Expression changes of target transcripts were quantified on root samples (three independent biological replicates) by quantitative real-time PCR (RT-qPCR). Roots from two plants from each treatment were pooled to form a biological replicate, immediately frozen in liquid nitrogen and stored at -80°C. Total RNA was isolated from each biological replicate and cDNA synthesis was performed as described in Chitarra et al. (2016). Genomic DNA contamination was checked before proceeding with the cDNA synthesis by PCR reactions using *LeEF* specific primers of tomato (**Supplementary Table S1**). RT-qPCR experiments were carried out in a final volume of 15 μl containing 7.5 μl of Rotor-Gene^TM^ SYBR^®^ Green Master Mix (Qiagen), 1 μl of 3 μM specific primers and about 10 ng of cDNA. Samples were run in the Rotor Gene apparatus (Qiagen) using the following program: 10 min pre-incubation at 95°C, followed by 40 cycles of 15 s at 95°C, and 30 s at 60°C. Each amplification was followed by melting curve analysis (60–94°C) with a heating rate of 0.5°C every 15 s. All reactions were performed with three technical replicates and only Ct values with a standard deviation that did not exceed 0.3 were considered. The comparative threshold cycle method (Rasmussen, 2001) was used to calculate relative expression levels using plant *LeEF* and *LeUBI* reference genes. Oligonucleotide sequences are listed in **Supplementary Table S1**. In detail, genes encoding for plant (*LePT1, LePT2, LePT3, LePT4*, and *LePT5*) and fungal (*RiPT* and *FmPT*) phosphate transporters have been considered.

#### Targeted Analysis of Leaf Volatiles

Leaf VOC analyses (isolation, identification and quantification) were done by headspace solid-phase microextraction-gas chromatography-mass spectrometry (HS-SPME-GC-MS). For sample preparation, 0.1 g of tomato leaf stored in a 2 ml screw cap headspace glass vial at -80°C were transferred at room temperature for 5 min and 0.5 ml of 30% NaCl solution containing the internal standard (2-hexanone at 1 ng/μl) was added to the vial. After mixing, samples were pre-incubated for 30 min at room temperature. A 65 μm PDMS/DVB SPME fiber (Supelco, Sigma Aldrich, Milan, Italy) was used for extraction procedure: the fiber was introduced into the vial headspace for 5 min.

A Hewlett Packard GC-MS system composed by a HP 5890 Series II Gas Chromatograph coupled to a HP 5971A Mass Selective Detector single quadrupole mass spectrometer was used. The GC-MS was equipped with a ZB-5MS column with column guard (Phenomenex) (30 m × 0.25 μm i.d.; film thickness 0.50 μm). Oven temperature was set up with following parameters: initial temperature 45°C maintained for 3 min, then to 220°C at 12°C/min, held isothermally at 220°C for 2.5 min. The volatiles trapped on the fiber were desorbed for 2 min at 250°C in the injection port of the GC; the injection was performed in splitless mode for 1 min. To prevent cross-contamination between successive samples, the fiber was cleaned by exposure in a different GC injection port for 5 min at 250°C before a new sampling process. Ultra high purity helium was used as the carrier gas at a constant head pressure of 12 psi (corresponding to 1 ml/min at 45°C). Transfer line temperature was 280°C.

Data were recorded in full scan mode from 40 to 550 m/z, using an electron ionization (EI, 70 eV) source, with 1.53 scan/s. Data acquisition and processing were performed using HP ChemStation software (version D.02.00).

Quantitative analysis by GC-MS was performed recording the signals in selected ion monitoring (SIM) mode; the areas of the quantifier ion peak of each analyte were measured and compared to that of the internal standard 2-hexanone. In **Supplementary Table S2** the retention time and principal ion of each compound are reported.

Calibration curves constructed with pure standards – trans-2-hexenal, methyl salicylate, eugenol, α–phellandrene, β–phellandrene (Sigma Aldrich) – in the ranges of the interest concentrations allowed the calculation of the compound concentrations in the samples.

### Experiment 2

Experiment 2 was devoted to evaluate the impact of AM symbiosis on a combined abiotic (moderate water stress) and biotic (aphid infestation) stress condition. To this aim, aphid survival (a measure of direct plant defense), parasitoid attractiveness (a measure of indirect defense) and VOC emissions were considered in unstressed and water stressed conditions. Considered treatments were: (i) AM fungus [non-colonized (Ctrl) and *R. intraradices*-colonized (Rin)], (ii) water stress [none (NS) and moderate (WS)], and (iii) Aphid infestation (Aph) and their combinations. In detail, for aphid survival, 20 plants non-colonized (Ctrl) and 20 colonized with *R. intraradices* (Rin) were prepared for the aphid survival experiment: 10 plants for each condition were maintained under a well water conditions (CtrlNS and RinNS) and 10 were subjected to a moderate water stress (WS and WS + Rin). By contrast, for parasitoid attractiveness and VOC collection, 6 plants for the following 8 conditions were considered: (NS, Aph, Rin, WS, Rin + Aph, WS + Rin, WS + Aph, and WS + Rin + Aph), totalling 48 plants.

#### Insect Rearings

Both the herbivore and its parasitoid were reared at CNR-Institute for Sustainable Plant Protection (Portici, NA, Italy) as follows:

– the potato aphid *Macrosiphum euphorbiae* Thomas (Hemiptera: Aphididae) was reared on tomato plants *cv*. San Marzano nano (i.e., dwarf) from material collected in the field (Scafati, SA, Italy) on the same plant cultivar in 2001 and periodically refreshed by adding field collected aphids. Rearing conditions were: 20 ± 1°C, 18 h light/6 h dark photoperiod, 65 ± 5% relative humidity.
– the aphid parasitoid *Aphidius ervi* Haliday (Hymenoptera: Braconidae) was permanently reared on the pea aphid *Acyrthosiphon pisum* reproduced on broad bean plants (*cv*. Aquadulce) from material collected in the field (Battipaglia, SA, Italy) in 2001 on alfalfa and periodically refreshed by adding field collected aphids. Rearing conditions were: 20 ± 1°C, 18/6 h light/dark photoperiod, 65 ± 5% relative humidity (Guerrieri et al., 2002).

#### Aphid Survival Assays

For each experimental condition 10 plants were infested with one newly born nymph of *M. euphorbiae*, transferred with a soft brush. All plants were checked daily to assess presence of aphids, presence of exuviae (evidence of molting), presence and number of newly laid nymphs and dead aphids. When reproduction started, the offspring were counted and removed daily. The mortality of the aphids was recorded daily until the death of the last individual. The parameters of the bioassay were set as indicated above.

#### Wind-Tunnel Bioassay

Plants were tested in a wind-tunnel bioassay for their attractiveness toward the aphid parasitoid wasp *A. ervi*. For the aphid infestation fifty mixed-aged individuals of *M. euphorbiae* (mimicking a natural population) were gently transferred by paintbrush on tomato leaves allowing them to feed for 1 week. For the double stress thesis, the aphids were added at the onset of water deficit imposition. These treatments were performed using both inoculated with *R. intraradices* and non-inoculated tomato plants. For each experimental condition, a total of 6 plants was used and offered individually every day for 6 consecutive days in a random order to reduce any bias related to the time of the experiments. One hundred parasitoid females were tested singly for each target in no-choice experiments, and observed for a maximum of 5 min. The percentage of response (oriented flights, landings on the target) to each target plant was calculated. The parameters of the bioassay were set as follows: 20 ± 1°C; 65 ± 5% relative humidity; 25 ± 5 cm s -1 wind speed; 50 cm distance between releasing vial and plant target.

#### VOC Collection and Analysis

Immediately after the bioassay in the wind tunnel, each plant was used for the collection of head-space volatiles by an airtight entrainment system. The system consisted of a bell jar containing a single potted plant and the jar was connected to a circulation pump forcing the air in an adsorbent trap made of Tenax TA, 60–80 mesh (Sigma-Aldrich). Each collection lasted 3 h under controlled conditions (24 ± 2°C, 18/6 h light/dark photoperiod, 70 ± 10% relative humidity, 700 μmol m 2 s 1 PPFD). Collected volatiles were analyzed by an integrated system including thermal desorber (Tekmar TD-800) mounted on a gas chromatograph (column: RTX-200, 60 m, 0.25 mm ID, 0.25 μm, carrier gas: He) coupled to a mass spectrometer detector. The resulting peaks were compared with a compound database library (National Institute of Standards and Technology) and the following available authentic standards: anisole-p-allyl, camphor, 3-carene, (E)-β-caryophyllene, chlorobenzene, α-copaene, α-cubebene, p-cymene, decane, p-dichlorobenzene, dodecene, eucalyptol, eugenol, (E)-β-farnesene, α-gurjunene, hexanal, (Z)-3-hexen-1-ol, humulene (=α-caryophyllene), (R)-(+)-limonene, (S)-(-)-limonene, linalool, (+)-longifolene, menthol, 6-methyl-5-hepten-2-one, methyl salicylate, b-myrcene, (Z)-nerolidol,(E)-β-ocimene, (R)-(-)-α-phellandrene, α-pinene, skatol, α-terpinene, γ-terpinene, α-terpineol, and terpinolene.

### Statistical Analysis

Analysis of variance (ANOVA) of the experimental data was performed using the SPSS software. When ANOVA indicated that either stress (stress: NS, MS, and SS) or mycorrhizal colonization (myco: Ctrl, Fmos, and Rin) or their interaction was significant, mean separation was performed using the Tukey HSD test, adopting a probability level of *P* < 0.05. The volatile emission patterns, measured as peak areas divided by fresh plant weight, were analyzed by Detrended Correspondence Analysis (DCA). Data were square root transformed to meet normal distribution. Analysis was conducted using the R statistics programming environment 3.3.3 (R Development Core Team, 2012) and the ‘vegan’ package ver. 2.5-2. Multivariate analysis of variance by permutation (PMANOVA) was used to test for significant differences between group centroids using vegan’s adonis functions (Oksanen et al., 2013). The number of parasitoids responding to each target was compared by a G-test for independence with William’s correction. The resulting values of G were compared with the critical values of *χ*2 (Sokal and Rohlf, 1995). To analyze the effect of each experimental condition on aphid survival, we used function survfit and Kaplan–Meier plot to visualize the survival curves, contained in the R package ‘survival’ version 2.41-3.

## Results

### Biometric Parameters and Ecophysiology

In our experiments, biometric parameters including plant height, the internodes/height ratio and shoot diameter have been affected by both water treatments and AM colonization, while no significant differences were registered for the chlorophyll content index (CCI; **Supplementary Figures S1A–D**). Plant height and the internodes/height ratio were influenced by stress (S) and myco (M) treatments with no significant S × M interaction, although Fmos-inoculated plants under MS and SS conditions showed higher height when compared with RinMS/SS and CtrlSS plants (**Supplementary Figure S1A** and **Table S3**). Considering the internodes/height ratio, highest values were recorded for RinMS and SS plants with respect to other treatments (**Supplementary Figure S1B** and **Table S3**). Conversely, shoot diameter did not show a different trend among the M variables, while a significant impact of the S variables and S × M interaction was observed (**Supplementary Figure S1C**).

**FIGURE 1:**
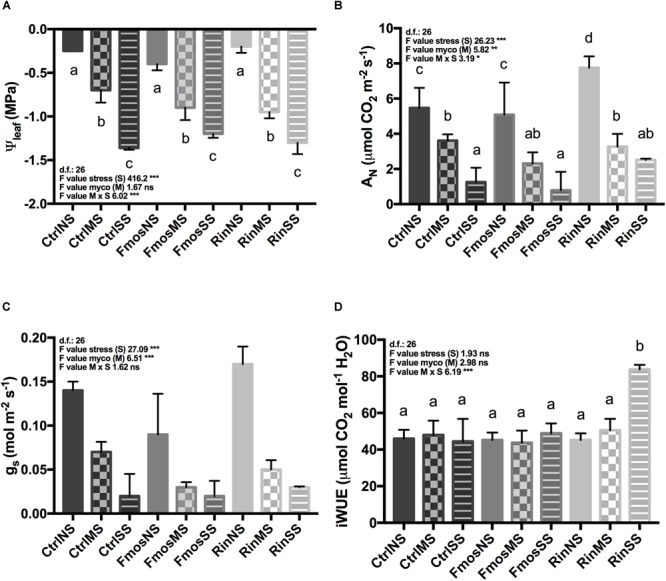
Leaf water potential and gas exchanges in AM– and AM+ plants unstressed (NS) and subjected to two different water deficit levels (moderate stress, MS; severe stress, SS), measured at the end of the experiment. **(A)** Leaf water potential (Ψ_leaf_); **(B)** net photosynthetic rate (A_N_); **(C)** stomatal conductance (g_s_); and **(D)** iWUE levels in AM– (Ctrl) and AM+ (Rin and Fmos) plants. All data are expressed as mean ± SE (*n* = 5). ns, ^∗, ∗∗^, and ^∗∗∗^: non-significant or significant at *P* ≤ 0.05, *P* ≤ 0.01, and *P* ≤ 0.001, respectively. Different letters above the bars indicate significant differences according to Tukey HSD test (*P* ≤ 0.05), considering S × M interaction. Analysis of variance on the single variables is reported in **Supplementary Table S3**.

The imposed water stress conditions differently affected leaf water potential (Ψ_leaf_) levels independently from controls and the AM inoculum used. At the end of the experiment, significant differences were observed. NS plants showed in fact values lower than/equal to -0.5 MPa, while Ψ_leaf_ values of about -0.9 MPa and -1.4 MPa were observed in MS and SS plants, respectively (**Figure 1A**).

Considering A_N_ rates, a decreasing trend was observed from NS to MS and SS conditions. Significant higher A_N_ rates were recorded in NS condition, particularly in RinNS plants. Conversely, significant lower values were measured under SS condition with the exception of RinSS plants that were statistically similar to FmosMS ones (**Figure 1B**). A similar trend was also evident for g_s_, although a significant influence was observed only for S and M variables (**Figure 1C** and **Supplementary Table S3**). In addition, iWUE index, irrespectively of treatment or water status, showed similar values, with the exception of RinSS plants where significant higher iWUE was observed (**Figure 1D**).

### Phosphorus Determination and Expression of Phosphate Transporter Genes

In no stress condition, no significant differences were detected among the three treatments in phosphorous (P) determination in leaves, i.e., CtrlNS, RinNS, and FmosNS plants, although higher values have been measured in AM-colonized plants in respect to non-colonized ones, with the highest P content (g/kg) recorded in *F. mosseae*-colonized plants. While no strong differences have been registered under a moderate stress condition (MS), under a severe water stress condition (SS) a higher leaf P content was detected in *F. mosseae*-colonized plants mainly respect to Ctrl plants (data not shown).

**FIGURE 2:**
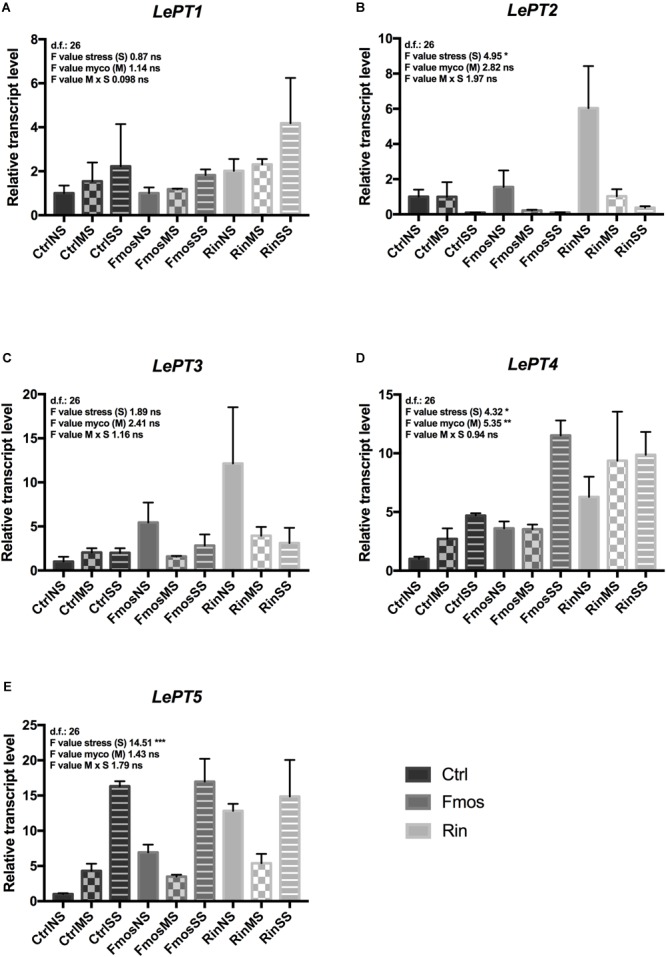
Expression changes of tomato phosphate transporter (PT) genes in roots of AM– and AM+ plants upon irrigation (NS), moderate water stress (MS), and severe water stress (SS) conditions. RT-qPCR analysis of **(A)** *LePT1*, **(B)** *LePT2*, **(C)** *LePT3*, **(D)** *LePT4*, and **(E)** *LePT5*. Lower letters denotes significant differences attested by Tukey HSD test (*P* < 0.05). All data are expressed as mean ± SE (*n* = 3). ns, ^∗, ∗∗, ∗∗∗^: non-significant or significant at *P* ≤ 0.05, *P* ≤ 0.01, and *P* ≤ 0.001, respectively. Different letters above the bars indicate significant differences according to Tukey HSD test (*P* ≤ 0.05), considering S × M interaction. Analysis of variance on the single variables is reported in **Supplementary Table S3**.

**FIGURE 3:**
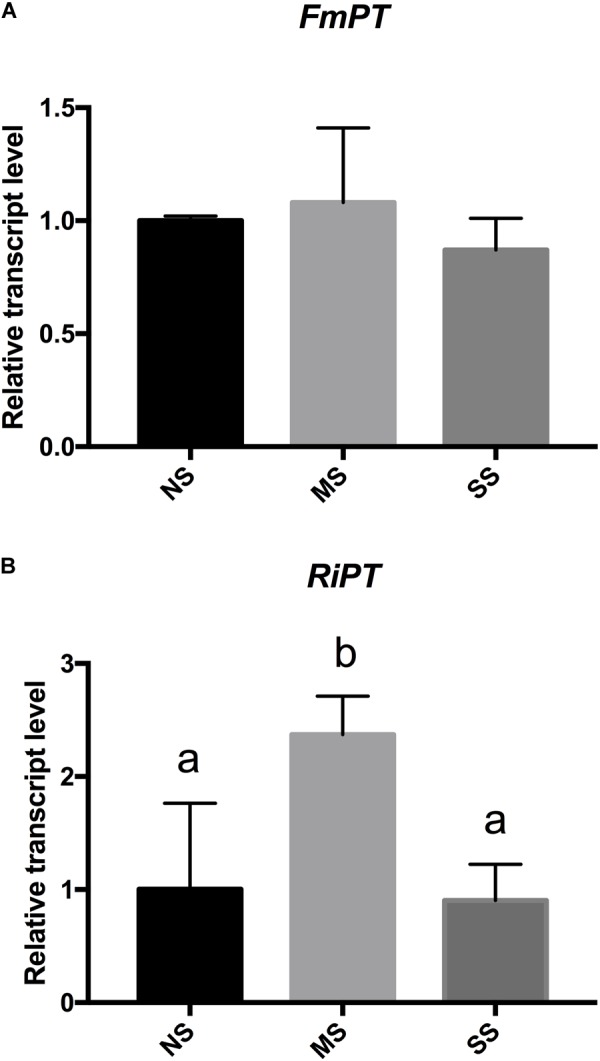
Expression changes of fungal phosphate transporter (PT) genes in AM+ roots upon irrigation (NS), moderate water stress (MS), and severe water stress (SS) conditions. RT-qPCR analysis of **(A)** *F. mosseae* PT (*FmPT*) and **(B)** *R. intraradices* PT (*RiPT*) genes, respectively. Lower letters denotes significant differences attested by Tukey HSD test (*P* < 0.05). Data are expressed as mean ± SE (*n* = 3).

Additionally, the expression of plant and fungal Pi transporter genes was evaluated in roots from plants at different growth conditions (no stress and water deficit). Expression of PT genes was not influenced by M x S interaction. In detail, although statistical analysis showed an effect of the S variable only for *LePT2*, both *LePT1* and *LePT2* changed their expression under water deficit, but with an opposite trend (**Figures 2A,B** and **Supplementary Table S3**). *LePT1* expression in fact increased under water deficit with the highest value in RinSS, while *LePT2* transcripts progressively decreased with the water deficit. Among the mycorrhizal-inducible PT genes (*LePT3, LePT4*, and *LePT5*), *LePT3* resulted to be overexpressed in RinNS roots, while decreased under water stress mainly in AM-colonized plants (**Figure 2C**). *LePT4* resulted to be influenced by S and M, while *LePT5* only by S (**Figures 2D,E** and **Supplementary Table S3**). In detail, these two last transporters showed a similar expression trend, with increased levels in SS plants (**Figures 2D,E**) with respect to the other treatments (NS and MS). However, *LePT4* resulted overexpressed in RinSS plants, while *LePT5* was in FmosSS ones, suggesting a species-specificity effect. From the fungal side, *F. mosseae* PT gene showed lower expression levels than *R. intraradices* PT in all the considered conditions (**Figures 3A,B**). Under water deficit, the expression of these fungal genes was not significantly affected, except for *RiPT* that is significantly up-regulated in MS (**Figure 3B**).

### Target Metabolomics

The target leaf VOCs present in NS and SS plants in the presence or in the absence of either *F. mosseae* or *R. intraradices* have been determined and quantified. The main VOCs, *trans*-2-hexenale, methyl salicylate, eugenol, α-phellandrene and β-phellandrene increased in SS, mainly in the presence of the AM fungus *F. mosseae*, in agreement with statistical analysis that showed a highly significant influence of both S and M variables for all the considered VOCs (**Table 1**). Differently, the S × M interaction resulted to be significant for all the metabolites with the exception of *trans*-2-hexenal (**Table 1**).

**Table 1:** Quantification of VOC products in tomato leaves.

Source of variance	Trans-2-hexenal	Methyl salicylate	Eugenol	α-Phellandrene	β-Phellandrene
Stress	^∗∗∗^	^∗∗∗^	^∗∗∗^	^∗∗∗^	^∗∗∗^
Myco	^∗∗∗^	^∗∗∗^	^∗∗∗^	^∗∗∗^	^∗∗∗^
Stress ^∗^ myco	ns	^∗^	^∗^	^∗∗∗^	^∗∗∗^
**Stress**					
NS	131.97 ± 47.41a	0.89 ± 0.55a	0.41 ± 0.06a	0.06 ± 0.10a	0.92 ± 0.76a
SS	160.81 ± 47.06b	1.41 ± 0.45b	0.52 ± 0.08b	0.16 ± 0.17b	2.00 ± 1.67b
**Myco**					
CTRL	127.61 ± 23.70a	1.09 ± 0.47a	0.44 ± 0.09a	0.05 ± 0.04a	0.80 ± 0.30a
Fmos	183.19 ± 64.00b	1.48 ± 0.74b	0.51 ± 0.11b	0.25 ± 0.17b	2.72 ± 1.77b
Rin	126.29 ± 20.32a	0.89 ± 0.17a	0.45 ± 0.06a	0.02 ± 0.02a	0.79 ± 0.22a
**Stress ^∗^ myco**					
CTRLNS	117.17 ± 16.08	0.69 ± 0.08a	0.37 ± 0.03a	0.04 ± 0.01a	0.58 ± 0.17a
CTRLSS	138.05 ± 26.38	1.499 ± 0.31bc	0.50 ± 0.08c	0.08 ± 0.05ab	1.01 ± 0.25ab
FmosNS	157.54 ± 76.79	1.19 ± 0.96ab	0.45 ± 0.06ab	0.23 ± 0.13b	1.42 ± 1.25ab
FmosSS	210.75 ± 21.96	1.82 ± 0.20c	0.60 ± 0.07d	0.38 ± 0.09c	4.15 ± 0.96ab
RinNS	125.54 ± 20.98	0.87 ± 0.19a	0.42 ± 0.06ab	0.07 ± 0.003a	0.75 ± 0.13b
RinSS	120.87 ± 16.46	0.94 ± 0.19a	0.47 ± 0.06bc	0.04 ± 0.004a	0.77 ± 0.24c

*All data are expressed as mean ± SD. Ns, ^∗, ∗∗, ∗∗∗^: non-significant or significant at *P* ≤ 0.05, *P* ≤ 0.01, and *P* ≤ 0.001, respectively. Different letters within each column indicate significant differences according to Tukey HSD test (*P* ≤ 0.05).*

### Insect Performance and VOC Emission

**FIGURE 4:**
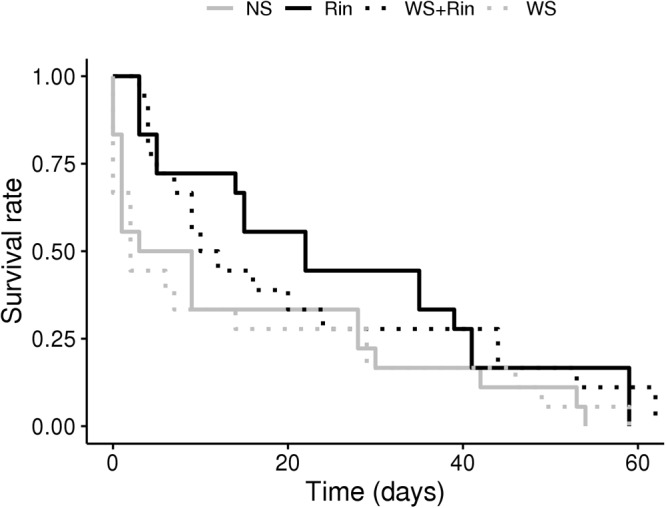
Probability of aphid *Macrosiphum euphorbiae* survival when fed on tomato AM– and AM+ plants (inoculated with R. intraradices), upon irrigation (NS and Rin) and water-stress (WS and WS + Rin) conditions.

Root colonization by *R. intraradices* significantly improved the development of *Macrosiphum euphorbiae* on tomato in both conditions tested (NS and WS). When aphid survival curves were calculated, survival probability proved to be higher on colonized plants (**Figure 4**, black lines, log-rank: colonized vs. non-colonized plants, *P* = 0.015). Conversely, water stress (dashed lines) did not affect the development of the aphids (**Figure 4**, log-rank: WS vs. NS, *P* = 0.87).

**FIGURE 5:**
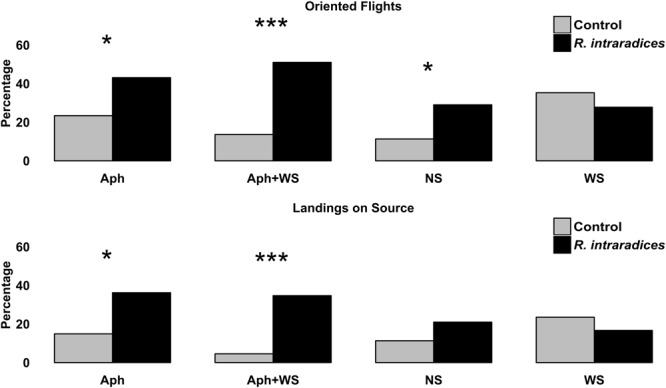
Flight behavior (oriented flights and landing on source) of *Aphidius ervi* females (%) toward tomato plants inoculated with *R. intraradices*, infested by aphids, their combination and relative control. Asterisks assigned by *G*-test for independence (^∗^*P* < 0.05 and ^∗∗∗^*P* < 0.001).

Root colonization by *R. intraradices* affected the foraging behavior of *A. ervi* in almost all conditions tested (**Figure 5**). Oriented flights significantly increased by root colonization in control, aphid infested and double stressed colonized plants. The same pattern was recorded for the landings on the source with the exception of control plants where root colonization did not affect parasitoid response. The presence of *R. intraradices* did not enhance the foraging behavior of *A. ervi* in condition of water stress.

**FIGURE 6:**
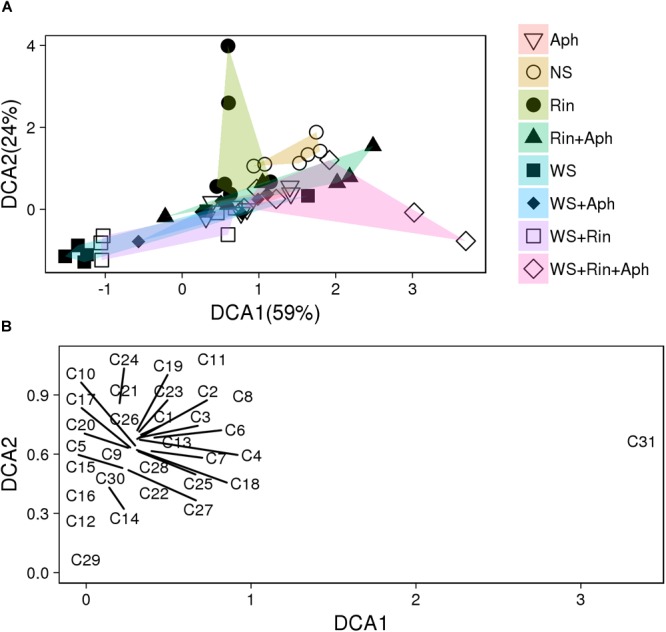
Detrended Correspondence Analysis (DCA) score **(A)** and loading **(B)** plots of the first two components based on volatiles emitted tomato plants that were not stressed (NS), aphid-attacked (Aph), water-stressed (WS), and exposed to both water stress and aphid infestation (WS + Aph). **(A)** Each experiment was carried out using uninoculated (NS, Aph, WS, and WS + Aph) and inoculated with *R. intraradices* (Rin, Rin + Aph, WS + Rin, and WS + Rin + Aph) plants. In brackets the percentage of variation explained is indicated. Multivariate analysis of variance by permutation was used to test for significant differences between group centroids (see **Supplementary Table S4**). **(B)** Loading plot of the DCA first two components, showing the contribution of each VOC (C1–C31, listed in **Table 2**) to the model.

Several of the collected volatiles were significantly affected by the presence of *R. intraradices* mainly in stressed plants (**Table 2, Figure 6**, and **Supplementary Table S4**). The DCA analysis separate plots on the basis of different treatments (**Figure 6** and **Supplementary Table S4**). Distance between centroids (**Supplementary Table S4** and **Figure 6**) indicated that volatile blends differ significantly in all treatments expect for (WS vs. Aph) and in almost all their combinations (double and triple, **Supplementary Table S4**). Whilst in non-stressed plants an increase was recorded in colonized plants in respect to non-colonized ones, in double stressed plants the AM fungus affected the VOCs emission with a significant decrease of most of volatiles, with the exception of methyl salicylate emission that increased under double stress conditions with respect to all other treatments (**Table 2**). Conversely, three terpenes (α-terpinene, α-phellandrene and β-phellandrene) were released at about half rate by *R. intraradices* colonized plants under double stress in respect to non-colonized ones.

**Table 2:** VOCs released by tomato plants that were not stressed (NS), aphid-attacked (Aph), water-stressed (WS), and exposed to both water stress and aphid infestation (WS + Aph).

	Mean value of VOC (ng g^-1^ fr wt) ± SE						Three way ANOVA effects					

	NS	Rin	Aph	Rin + Aph	WS	WS + Rin	WS + Aph	WS + Rin + Aph	Rin (1)	Aph (2)	WS (3)	1 × 2	1 × 3	2 × 3	1 × 2 × 3
2,4-Dimethyl-1-heptene	242.2 ± 26.2	353.3 ± 29.8	285.7 ± 30.6	392.7 ± 18.9	258.2 ± 41.7	276.7 ± 69.5	414.8 ± 35	236.2 ± 44.8					**^∗∗^**		
Ethylbenzene	57.2 ± 3.8	74 ± 7.8	72.5 ± 3.4	73.2 ± 4.4	64.2 ± 8.4	75.3 ± 9.5	77.8 ± 7.0	53.3 ± 9.9				**^∗^**			
p-Xylene	242.2 ± 26.2	353.3 ± 29.8	285.7 ± 30.6	392.7 ± 18.9	258.2 ± 41.7	276.7 ± 69.5	414.8 ± 35	236.2 ± 44.8				**^∗^**			
o-Xylene	99.7 ± 9.2	144.8 ± 15.6	152.3 ± 10.6	124.8 ± 10.7	114.8 ± 26.6	120.3 ± 24.0	95.8 ± 26.5	100.7 ± 23.3							
α-pinene	134.8 ± 77.3	186 ± 106	236.3 ± 143.7	88.2 ± 7.7	158.5 ± 36	148.3 ± 36.9	153.2 ± 29.8	70.7 ± 18.2							
2-Ethylhexanal	24.2 ± 4.2	34.5 ± 3.2	19.5 ± 2.5	37.7 ± 3.5	17.7 ± 3.5	18 ± 3.8	36.8 ± 6.5	28 ± 9.1					**^∗^**		
4-Methylnonane	15.8 ± 2.3	39.8 ± 8.5	23.3 ± 3.6	28.7 ± 5.9	14.2 ± 3.1	15.2 ± 8.1	33.5 ± 4.9	12 ± 4.8			**^∗^**		**^∗∗^**		
Benzene, 1-ethyl-3-methyl-	24.2 ± 4.0	25.7 ± 9.2	11.8 ± 5.6	8.3 ± 5.3	2.7 ± 2.7	4.2 ± 4.2	17.5 ± 8.2	22.7 ± 7.6						**^∗∗^**	
Benzaldehyde	72.8 ± 4.1	98.5 ± 6.4	104 ± 7.4	96.5 ± 22.2	104.7 ± 18.2	111 ± 19.8	135 ± 12.3	80.3 ± 17.8				**^∗^**			
1-Decene	151 ± 7.3	200.8 ± 7.8	203.8 ± 15.6	238.8 ± 27.3	183.2 ± 16.4	222.2 ± 40.5	254.8 ± 27.5	174.5 ± 43.6							
Benzene, 1,3,5-trimethyl-	89.3 ± 7.3	113.7 ± 8.6	93.3 ± 3.9	157.2 ± 50.8	18.5 ± 11.9	55.8 ± 29.7	94.7 ± 20.5	86.8 ± 20.1		**^∗∗^**	**^∗∗∗^**			**^∗^**	
α-Terpinene	185.7 ± 15.5	452.5 ± 36.3	874.8 ± 133.3	603.2 ± 196.1	1387.8 ± 288.5	1583.7 ± 442.4	1155.3 ± 248.4	490.3 ± 179.9			**^∗∗∗^**	**^∗∗^**		**^∗∗∗^**	
α-phellandrene	84.3 ± 3.5	128.8 ± 13	149.7 ± 33.7	154.5 ± 15.6	50 ± 26.9	295.7 ± 156.7	241.8 ± 61.2	101 ± 24.7				**^∗^**			
1,4-dichlorobenzene	24.7 ± 1.3	33.7 ± 7.9	53.3 ± 6.2	92.7 ± 30.9	79.7 ± 25.0	108.3 ± 48.1	100.2 ± 26.3	36 ± 8.4						**^∗^**	
Limonene	384.7 ± 50.9	880 ± 67.6	1435.8 ± 198.4	1232 ± 406.7	2646 ± 612.7	2704.5 ± 555.7	2167.3 ± 269.2	1381 ± 357.2			**^∗∗∗^**	**^∗^**		**^∗∗^**	
β-phellandrene	233.3 ± 49.2	511 ± 46.3	1012.7 ± 97	800 ± 197.3	1407.7 ± 332.6	1409.7 ± 361.8	1424 ± 233.5	579.2 ± 162.8			**^∗∗∗^**	**^∗∗^**		**^∗∗∗^**	
Acetophenone	27.7 ± 1.9	39.3 ± 3.0	38.5 ± 1.4	47.7 ± 5.7	40.8 ± 7.8	48.2 ± 12.4	56.8 ± 4.2	30.8 ± 5.2					**^∗^**		
p-Tolualdehyde	151.7 ± 17.1	218.5 ± 26.2	131.7 ± 23.5	236 ± 32.8	178 ± 28.2	204.7 ± 47.3	334.3 ± 139.7	84.2 ± 22.6					**^∗∗^**		**^∗^**
Methyl benzoate	21.5 ± 1.9	35.3 ± 6.7	25.5 ± 3.0	30 ± 4.7	24 ± 5.8	37.3 ± 6.8	41.8 ± 3.8	27.7 ± 4.0				**^∗∗^**			
Nonanal	60 ± 6.0	98.7 ± 9.1	92.5 ± 8.8	102.7 ± 17.4	89 ± 13.7	131.3 ± 25.2	164.2 ± 12.4	101.2 ± 19.7			**^∗^**	**^∗∗^**			
Camphor	19.7 ± 2.0	21.3 ± 4.7	23.7 ± 1.2	21.7 ± 4.8	18.3 ± 3.3	32.3 ± 5.3	46.8 ± 10.4	64 ± 40.9							
(Z)-3-Nonen-1-ol	2.2 ± 2.2	18.5 ± 0.4	15.3 ± 4.9	15.2 ± 4.8	9.8 ± 4.4	20.5 ± 7.2	25.3 ± 1.5	9.5 ± 6.0				**^∗∗^**	**^∗^**		
Naphthalene	410.3 ± 21.9	490.2 ± 21.1	473.2 ± 50.5	579 ± 52.3	350 ± 81.6	549.2 ± 63.9	712.3 ± 37.6	456.3 ± 100.3		**^∗^**		**^∗^**			**^∗∗^**
Methyl salicylate	15.2 ± 2.8	30.2 ± 1.5	105 ± 2.9	138.3 ± 7.6	41 ± 3.2	65.5 ± 8.3	188.7 ± 5.4	236.2 ± 14.5	**^∗∗∗^**	**^∗∗∗^**	**^∗∗∗^**			**^∗^**	
1-dodecene	1028.5 ± 49.1	1184.7 ± 48.3	1192.8 ± 117.8	1337.8 ± 189.3	1072.8 ± 71.6	1288.2 ± 174.3	1496.2 ± 100.5	885.5 ± 281.8							
Decanal	151 ± 7.3	200.8 ± 7.8	203.8 ± 15.6	238.8 ± 27.3	183.2 ± 16.4	222.2 ± 40.5	254.8 ± 27.5	174.5 ± 43.6							
2,5-Dimethylbenzaldehyde	13 ± 2.8	26 ± 2.1	21.5 ± 2.7	23 ± 3.5	21.3 ± 4.5	31.8 ± 5.0	41.5 ± 2.2	21.3 ± 7.7				**^∗∗^**			
Benzothiazole	35.2 ± 8.8	64.7 ± 6.5	51 ± 11.9	76.2 ± 9.7	50.2 ± 10.1	51 ± 13.3	85.0 ± 7.1	50.2 ± 15.0					**^∗∗^**		
Cumin aldehyde	3.7 ± 3.7	26.5 ± 8.0	8.5 ± 5.7	13.3 ± 9.2	36.3 ± 12.0	56.7 ± 12.0	40.5 ± 11.9	13.5 ± 6.4			**^∗∗∗^**	**^∗^**			
β-caryophyllene	93.3 ± 15.5	157 ± 33.8	196 ± 31.3	167.8 ± 45.3	156.2 ± 41.4	160.2 ± 15.1	190.8 ± 45.5	119 ± 41.6				**^∗^**			
O-cymene	0 ± 0	13.3 ± 8.5	0 ± 0	0 ± 0	0 ± 0	0 ± 0	0 ± 0	0 ± 0							

*Each experiment was carried out using uninoculated (NS, Aph, WS, and WS + Aph) and inoculated with *R. intraradices* (Rin, Rin + Aph, WS + Rin, and WS + Rin + Aph) plants. Asterisks indicate statistically significant effects tested by ANOVA (^∗^*P* < 0.05, ^∗∗^*P* < 0.01, ^∗∗∗^*P* < 0.001).*

## Discussion

Although several papers were dedicated to describe the effect of AM symbiosis on tomato response to water deficit (Dell’Amico et al., 2002; Subramanian et al., 2006; Aroca et al., 2008; Wang et al., 2014; Chitarra et al., 2016; Ruiz-Lozano et al., 2016; Rivero et al., 2018), here we have focused on some specific aspects less considered so far. In this study, we have examined the impact of AM symbiosis on the tomato response to water deficit considering some belowground traits related to symbiosis, i.e., the expression of Pi transporter genes, and some aboveground responses, in terms of plant performance traits and changes in metabolites having a role in tolerance/defense. Two different AM fungal species have been used, and results here presented confirmed a species-specific impact on belowground/aboveground interactions in tomato. Previous works already showed the species-specificity of the interactions between plants and AM fungi under stressed conditions (Chitarra et al., 2016; Quiroga et al., 2017; Pollastri et al., 2018). More recently, Rivero et al. (2018) reported an untargeted metabolomic analysis in tomato roots colonized by three AM fungi of different genera, to verify their impact on tomato tolerance to drought or salt stress. Plant growth responses were also considered and, overall, results showed that whilst some responses were common to all AM fungi tested, others were specifically related to single isolates. The common effect of all AM fungal species tested was a higher biomass (both above- and belowground) in respect to non-colonized plants, correlated with the degree of the stress. Here, tomato plants inoculated with *R. intraradices* showed higher internodes/height ratios, calculated as a measure of plant growth, under water deficit with respect to the other conditions. This result suggests a positive influence of this isolates in regimes of water deficit, probably due to a more compact plant architecture, and thus less subject to water dispersion. As confirmed by statistical analysis (**Supplementary Table S3**), no significant impact on the considered growth parameters has been recorded in tomato plants colonized by *F. mosseae*. The discrepancy with the paper by Rivero et al. (2018) could be due to the different growth conditions and most probably to a different tomato cultivar. A different impact of the two fungi on plant performance under water deficit can be also highlighted, with *R. intraradices* that leads to higher iWUE, as already reported in Chitarra et al. (2016). Overall, these results confirmed the importance to produce *ad hoc* formulates of AM fungi in relation to a specific plant species/cultivar and environmental condition, for a better exploitation/protection in the field (Berruti et al., 2013, 2015).

### Two AM Fungal Species Differently Affect Pi Uptake

Limited water resources and increasing soil salinity affect crop performance and productivity worldwide. These abiotic factors can be also accompanied by the low availability of many mineral nutrients including phosphorous (P) (Grattan and Grieve, 1999) and by the inhibition of mineral nutrient uptake and translocation (Wang et al., 2017). P fertilization can increase stress tolerance and productivity in several plant species and it appears mandatory to understand the mechanism/s of Pi acquisition and utilization under salinity and drought, to improve PUE and consequently crop productivity under abiotic stresses (Sawers et al., 2017; Wang et al., 2017). AM symbiosis promotes an improved nutritional status of the host plant (Smith and Smith, 2012), particularly under low nutrient availability. The success in nature of this symbiosis, known for more than 80% terrestrial plants, stays on the bidirectional nutritional exchanges, where the fungus supplies the plants with mineral nutrients and, in turn, receives carbon compounds (Balestrini and Lumini, 2018). Pi uptake, transfer and delivery have been largely investigated in AM roots, resulting in the characterization of a symbiotic Pi uptake pathway (Bucher, 2007; Javot et al., 2007; Smith and Smith, 2011). Particularly, PHT1 transporters, which are mainly involved in Pi uptake from soil and translocation, have been reported to be involved in the acquisition of Pi from AM (Glassop et al., 2005; Javot et al., 2007; Tamura et al., 2012; Yang et al., 2012; Duan et al., 2015; Liu et al., 2016; Tian et al., 2017; Liu et al., 2018). In tomato, eight PHT1 genes are expressed in the roots (Chen et al., 2014) and mycorrhizal-induced PT genes have been identified (Nagy et al., 2005, 2009). Different PT genes can be differentially regulated by drought. For example, several genes encoding Pi transporters from poplar and apple were found to be regulated by drought stress and Pi levels. These genes, especially those up-regulated by drought stress at low Pi level, might contribute to drought tolerance of crops in Pi-limited soils (Zhang et al., 2016).

Here, new information on the expression of five tomato PT genes in presence/absence of the AM fungus and in response to two different water deficit conditions (moderate and severe) is reported. As reported in Chitarra et al. (2016), root colonization seemed not to be strongly affected by a severe water stress in this tomato cultivar, independently of the AM fungal species considered. It is worth noting that the proportion of AM root colonization was greater for *R. intraradices*, while the percentage of arbuscules in the colonized portion was significantly greater for *F. mosseae*, both in NS and SS conditions, suggesting a different colonization strategy adopted by the two fungi. Looking at the regulation of PT genes, a significant up-regulation for the AM-inducible *LePT3, LePT4*, and *LePT5* has been observed in well-watered conditions, in agreement with previous data (Nagy et al., 2005, 2009; Balestrini et al., 2007). Under water stress, our results showed a different regulation of the considered PT genes. Particularly, it is worth noting the opposite trend for the two genes involved in the direct Pi uptake from soil (*LePT1* and *LePT2*), independently from the presence of the AM fungus. The poplar PHT1;2 has been already reported as induced by drought stress independently from Pi level (Zhang et al., 2016). Among the AM-inducible PT genes, *LePT3* seems not involved in response to water deficit at least in non-colonized plants as indicated by the lowest transcript level recorded in NS, whilst in colonized plants its expression fell independently by the stress level. *LePT4* and *LePT5* transcript levels both increased under water deficit with highest values in FmosSS, in agreement with the higher P content found in the leaves of these plants in respect to control ones. Overall results suggest a major role played by *LePT2* and *LePT4* in promoting tolerance to water deficit, particularly under a severe condition, although this is dependent from the fungal species. Since PT gene induction is also related to Pi-starvation, it is important to note that, in our experimental system, MS plants still received some nutrients during watering, while the nutritional status of SS plants did not received any external inputs even if the P content in leaves does not show a starvation level. We can then speculate that the transcript level for *LePT5* is more affected by a lower Pi content in soil than to water deficit. In fact, it has been already reported that the expression of some PT genes under drought can be Pi-dependent (Zhang et al., 2016). However, we cannot exclude a relation between our results on PT gene expression and the root architecture modification, which might happen under the different treatments. For example, it has been reported that in legumes phosphate starvation induced a higher number of lateral roots and *PT4* genes, homologs of *LePT4*, can play a role in root tips, creating a link among Pi-perception, root branching, and Pi-signaling mechanisms (Volpe et al., 2013, 2016).

The different behavior of the two AM fungal species tested further highlights the species-specificity of these complex interactions, regulating their final outcome, depending on both fungal and plant species considered (Sawers et al., 2017).

### AM Symbiosis Impacts Aphid Survivorship and VOCs Under Water Deficit and Tomato Response to Multiple Stresses

Root-associated microorganisms can alter the development of phytophagous insects in different ways. They can promote defensive responses that hamper the subsequent development of the invasive insect (Guerrieri et al., 2004) or, conversely, by improving plant quality that can enhance plant infestation (Battaglia et al., 2013). In our experiment, *R. intraradices* promoted the survival of *M. euphorbiae* in both control and water stressed plant. The null impact of water stress on aphid survival in *R. intraradices* colonized plants indirectly demonstrates the role of this AM species on plant tolerance to water deficit. Indeed, while water deficit should have had a deep impact on the survival of the sap feeding insects, by reducing the feeding time on sieve elements (Nachappa et al., 2016), this was not recorded for the aphid *M. euphorbiae* in our tests.

Plants synthetize and emit a large variety of VOC that function as important protective and signaling molecules (Guerrieri, 2016). Both biotic and abiotic stresses can induce plant volatile emission (Loreto and Schnitzler, 2010; Dong et al., 2016; Catola et al., 2018), including emissions of lipoxygenase (LOX) pathway products (various C6 aldehydes such *trans*-2-hexenale), shikimate pathway products (e.g., methyl salicylate), specific mono- and sesquiterpenes (such as β-phellandrene) and methanol (Niinemets, 2010; Dong et al., 2016). Additionally, the rate of emissions induced by a specific stress depends on genotype stress tolerance, timing, duration and severity of the stress (Niinemets, 2010; Copolovici et al., 2011; Niederbacher et al., 2015). Our target metabolomics analysis was focused on the impact of AM symbiosis on plant tolerance to a severe water stress, to evaluate an extreme condition that has been still less considered. The data underlined that the SS has an impact on the VOC production, demonstrating that plants, both non-colonized and *F. mosseae*-colonized, showed the highest concentration of the main VOC products with respect to the NS plants. Furthermore, a different effect of the AM fungal species was again evident, with *R. intraradices*-inoculated plants that showed similar level of all targeted metabolites in comparison with NS plants, suggesting an enhanced tolerance to this type of stress promoted by this AM fungal species. In tomato, a vast array of VOCs was found to be emitted under biotic (Digilio et al., 2010) and abiotic stresses, including water deficit (Catola et al., 2018), flooding (Copolovici and Niinemets, 2010), cold and heat (Copolovici et al., 2012; Kask et al., 2016). In our experimental study, where necessarily a MS has been used to permit the combined test with aphids, the emission of a typical tomato VOC bouquet under NS and water deficit conditions was determined. A change in VOC emission was particularly observed between non-colonized and *R. intraradices*-colonized plants both in well-watered condition and under a double stress (water deficit plus the aphid attack). However, the trend emission was opposite between NS and double stress plants. In NS plants colonized by *R. intraradices*, an increase in the release of VOC has been recorded particularly for the methyl salicylate. Significant changes in VOC emission has been registered for the single stress conditions in AM-colonized plants, while the presence of the AM fungus in double stressed plants lead to a decreasing trend in the emission of several compounds with one exception: methyl salicylate. This result strongly supported the behavioral observations given that methyl salicylate plays a major role in the attraction of *A. ervi* (Sasso et al., 2007), being detected by this parasitoid at antennal level at a concentration as low as 0.01 gr/ml (Sasso et al., 2009). On the other hand, the strong reduction in the release of the terpenes in double stressed plants colonized by *R. intraradices* in respect to non-colonized ones could be due to a shift toward the production of non-volatile compounds (essential isoprenoids such as carotenoids) in respect to VOC biosynthesis (not essential isoprenoids). This has been already reported by Asensio et al. (2012) that found that water stressed colonized plants plus exogenous jasmonic acid (JA) treatment (mimicking a wound) have a higher level of carotenoids, which have an important role in photosynthesis, with respect to non-colonized plants. The reduction of monoterpenes recorded in our experiment is also in line with the data of these same authors that explained such a reduction with the high demand of carbon by the AM fungus, particularly under combined stress (drought + JA treatment) in respect to unstressed and non-colonized plant (Asensio et al., 2012). However, a correlation between the VOC emission in colonized plants under double stress and a possible better P availability in the same plants, cannot be excluded, considering that nutritional status might have an impact on plant isoprenoid production (Asensio et al., 2012).

The effect of *R. intraradices* on plant resistance toward aphids resulted bifaceted. AM-colonized plants represented a better substrate for the survival aphid *M. euphorbiae* in respect to non-colonized ones thus reducing plant direct resistance toward insect pests. This negative effect on plant fitness was largely compensated by the significant enhancement of indirect defense as represented by the significant higher attraction of the parasitoid *A. ervi* recorded for colonized plants in respect to non-colonized ones. The increased level of indirect defense persisted in *R. intraradices* colonized plants also in conditions of double stress and was supported by the release of key attractive compounds (methyl salicylate).

## Conclusion

Our results suggest that the two AM fungi can trigger different adaptation strategies against environmental stresses, with *F. mosseae* that seems more effective on VOC production and *R. intraradices* on the considered plant performance traits, e.g., leading to a significant higher water use efficiency under a severe water stress. Additionally, *R. intraradices* was demonstrated to be effective on response to combined abiotic and biotic stress, the latter in terms of attractiveness toward the natural enemies of aphids. However, although AM fungi enhance crop tolerance to environmental stresses, the exploitation and the practical application in the field of these symbionts require a thorough identification of the mechanisms involved in nutrient transfer, the metabolic pathways induced by single and multiple stresses and the physiological mechanisms leading to improved tolerance. Our results well fit with this aim, adding new tiles to the pieces of information already present about the mechanisms involved in the tolerance to water and biotic stresses in tomato during AM symbiosis, and in nutrition aspects such as Pi uptake. Overall, our results confirmed the importance to develop mixed AM-based products, depending on the environmental conditions to be faced, choosing the right symbiotic partner/s (i.e., plant cultivar, AM fungus) that lead to different outcomes in terms of plant tolerance to stress. This is particularly urgent in a scenario of climate change as characterized by a progressive lack of water that combines, in the field, with other abiotic (e.g., nutritional availability and salinity) and biotic (e.g., insects and pathogens) stresses. However, considering the species-specificity in affecting tolerance/resistance traits, it is relevant to test individually an AM species/isolate before to produce a mixed inoculum, to avoid a null effect on plant response.

## Author Contributions

RB, EG, and BM conceived and designed the experiment. VV and WC performed the molecular analyses and the eco-physiological measurements. PC performed the multiple stress experiment and VOC collection. MV performed the phosphate determination. WC and PC performed the statistical analysis for all the data. PB participated to the metabolite extraction. GM, GP, CDS, and BM performed the target metabolomics experiment. EG participated to the multiple stress experiment and analyzed the VOC data. RB prepared the plants and performed the molecular analyses with VV. RB and VV wrote the manuscript with the contribution of EG and WC. All authors read and approved the manuscript.

## Conflict of Interest Statement

The authors declare that the research was conducted in the absence of any commercial or financial relationships that could be construed as a potential conflict of interest.

## Footnotes

1 http://www.mycagrolab.com/

## Supplementary Material

FIGURE S1 **(A–C)** Biometric parameters and **(D)** chlorophyll content index (CCI) and of AM- and AM+ tomato plants upon unstressed and water stressed (MS and SS) conditions. All data are expressed as mean ± SD. ns, ^∗, ∗∗, ∗∗∗^: non-significant or significant at *P* ≤ 0.05, *P* ≤ 0.01, and *P* ≤ 0.001, respectively. Different letters above the bars indicate significant differences according to Tukey HSD test (*P* ≤ 0.05), considering S × M interaction. Analysis of variance on the single variables is reported in **Supplementary Table S3**.

TABLE S1 List of the oligonucleotides used in this study.

TABLE S2 Retention time and principal ion of each compound.

TABLE S3 Analysis of variance on single variables referred to **Figures 1, 2** and **Supplementary Figure S1**.

TABLE S4 Results of pairwise multivariate analysis of variance by permutation among VOCs released by tomato plants (see **Table 2** for treatments). Significant values are in bold.
