# Supplementary figures and images for: The Association With Two Different Arbuscular Mycorrhizal Fungi Differently Affects Water Stress Tolerance in Tomato
Source: Front Plant Sci. 2018 Oct 9;9:1480. doi: 10.3389/fpls.2018.01480 (PMC6189365; doi:10.3389/fpls.2018.01480)

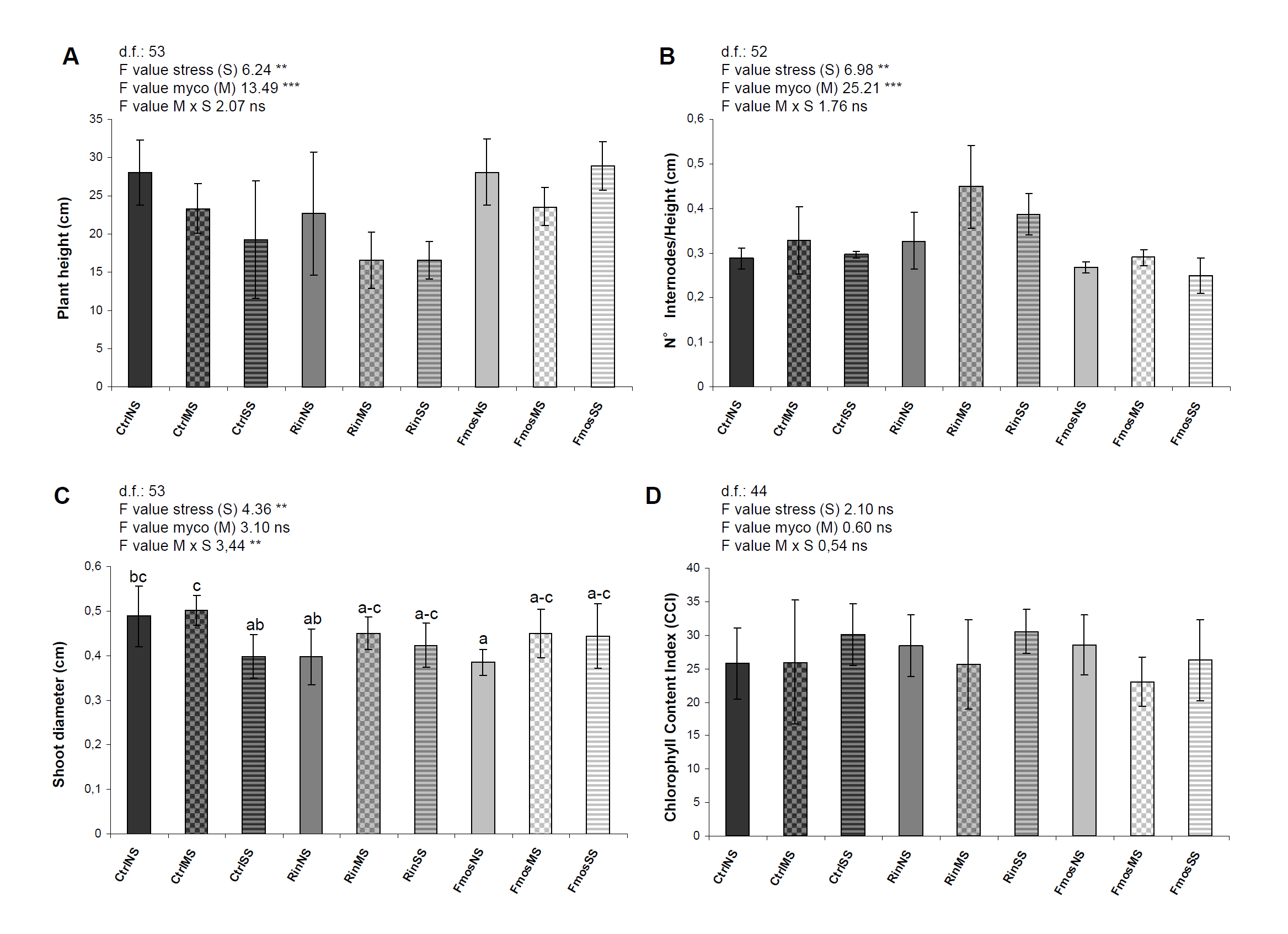

Supplement: FIGURE S1 — (A–C) Biometric parameters and (D) chlorophyll content index (CCI) and of AM- and AM+ tomato plants upon unstressed and water stressed (MS and SS) conditions. All data are expressed as mean ± SD. ns, ∗, ∗∗, ∗∗∗: non-significant or significant at P ≤ 0.05, P ≤ 0.01, and P ≤ 0.001, respectively. Different letters above the bars indicate significant differences according to Tukey HSD test (P ≤ 0.05), considering S × M interaction. Analysis of variance on the single variables is reported in Supplementary Table S3. [file Image_1.TIF]
