# Supplementary material for: The Association With Two Different Arbuscular Mycorrhizal Fungi Differently Affects Water Stress Tolerance in Tomato
Source: Front Plant Sci. 2018 Oct 9;9:1480. doi: 10.3389/fpls.2018.01480 (PMC6189365; doi:10.3389/fpls.2018.01480)
Supplement: TABLE S1 — List of the oligonucleotides used in this study. [file Table_1.DOCX]

**Table S1.** List of the oligonucleotides used in this study.

| **Primer_ID** | **Primer sequence (5’-3’)** |
| --- | --- |
| *LeEF_F* | CTCCATTGGGTCGTTTTGCT |
| *LeEF_R* | GGTCACCTTGGCACCAGTTG |
| *LeUbi_F* | CTTGTTGGGGTAATCCTCAG |
| *LeUBi_R* | ACGAGAGAACACAAAGCACA |
| *LePT1_F* | TTCCTCTTCCCCTTCGTTTTC |
| *LePT1_R* | TTCCTCGGTATGCTGTTCAC |
| *LePT2_F* | ACAATAACACACTTGGCCAC |
| *LePT2_R* | AACTGGAGAAGCATGAGATC |
| *LePT3_F* | TCAATCTACTGATCCATCTAAAGTC |
| *LePT3_R* | TAGTTTGTGCATTTTCCCCTTTAG |
| *LePT4_F* | CGGGCAGAATGAGACACAGATG |
| *LePT4_R* | TGAACATAGAAAGCACAAGGCGTAGT |
| *LePT5_F* | GCAGAACGAGACGCAGATGAA |
| *LePT5_R* | TGCTGAATTTGATAAACTTGCCAA |
| *RiTEF_F* | GCTATTTTGATCATTGCCGCC |
| *RiTEF_R* | TCATTAAAACGTTCTTCCGACC |
| *FmosEF_F* | GCAGAACGTGAGCGTGGTAT |
| *FmosEF_R* | ACCAGTACCGGCAGCAATAA |
| *RiPT_F* | AACACGATGTCAACAAAGCAAC |
| *RiPT_R* | AAGACCGATTCCATAAAAAGCA |
| *FmPT_F* | ACTGTTGGCGCTTAGTGCTTGG |
| *FmPT_R* | CAGCCCAACTTGATTTTGGTACG |
