# Supplementary material for: The Association With Two Different Arbuscular Mycorrhizal Fungi Differently Affects Water Stress Tolerance in Tomato
Source: Front Plant Sci. 2018 Oct 9;9:1480. doi: 10.3389/fpls.2018.01480 (PMC6189365; doi:10.3389/fpls.2018.01480)
Supplement: TABLE S2 — Retention time and principal ion of each compound. [file Table_2.DOCX]

**Table S2.** Retention time and principal ion of each compound

| Compounds | Retention time (min) | Molecular Weight | Quantifier and qualifier ion (m/z) |
| --- | --- | --- | --- |
| Trans-2-hexenal | 6.65 | 98 | 69.83 |
| α-phellandrene | 9.43 | 136 | 93.136 |
| β-phellandrene | 9.86 | 136 | 93.136 |
| Methyl salicylate | 12.35 | 152 | 120.152 |
| Eugenol | 14.42 | 164 | 164.103 |
