# Supplementary material for: The Association With Two Different Arbuscular Mycorrhizal Fungi Differently Affects Water Stress Tolerance in Tomato
Source: Front Plant Sci. 2018 Oct 9;9:1480. doi: 10.3389/fpls.2018.01480 (PMC6189365; doi:10.3389/fpls.2018.01480)
Supplement: TABLE S3 — Analysis of variance on single variables referred to Figures 1, 2 and Supplementary Figure S1. [file Table_3.DOCX]

**Table S3.** Analysis of variance on single variables referred to Figure 1, 2 and S1.

| **Source of Variance** | **Figure 1A (Ψ_leaf_)** | **Figure 1B**  **(A_N_)** | **Figure 1C**  **(g_s_)** | **Figure 1D**  **(iWUE)** |
| --- | --- | --- | --- | --- |
| **stress** |  |  |  |  |
| NS | a | b | b | ns |
| MS | b | a | a | ns |
| SS | c | a | a | ns |
| **myco** |  |  |  |  |
| CTRL | ns | b | b | ns |
| Rin | ns | b | ab | ns |
| Fmos | ns | a | a | ns |

|  | **Figure 2A (*LePT1*)** | **Figure 2B**  **(*LePT2*)** | **Figure 2C**  **(*LePT3*)** | **Figure 2D**  **(*LePT4*)** | **Figure 2A (*LePT5*)** |
| --- | --- | --- | --- | --- | --- |
| **stress** |  |  |  |  |  |
| NS | ns | b | ns | a | a |
| MS | ns | ab | ns | ab | a |
| SS | ns | a | ns | b | b |
| **myco** |  |  |  |  |  |
| CTRL | ns | ns | ns | a | ns |
| Rin | ns | ns | ns | b | ns |
| Fmos | ns | ns | ns | ab | ns |

|  | **Figure S1A (Plant height)** | **Figure S1B**  **(N° Internodes/height)** | | **Figure S1C**  **(Shoot ∅)** | **Figure S1D**  **(CCI)** |
| --- | --- | --- | --- | --- | --- |
| **stress** |  |  |  | |  |
| NS | b | a | a | | ns |
| MS | a | b | b | | ns |
| SS | a | a | a | | ns |
| **myco** |  |  |  | |  |
| CTRL | b | a | ns | | ns |
| Rin | a | b | ns | | ns |
| Fmos | b | a | ns | | ns |
