# Supplementary material for: The Association With Two Different Arbuscular Mycorrhizal Fungi Differently Affects Water Stress Tolerance in Tomato
Source: Front Plant Sci. 2018 Oct 9;9:1480. doi: 10.3389/fpls.2018.01480 (PMC6189365; doi:10.3389/fpls.2018.01480)
Supplement: TABLE S4 — Results of pairwise multivariate analysis of variance by permutation among VOCs released by tomato plants (see Table 2 for treatments). Significant values are in bold. [file Table_4.DOCX]

**Table S4.** Results of pairwise multivariate analysis of variance by permutation among VOCs released by tomato plants (see Table 2 for treatments).

| **Comparison** | **d.f.** | **F Model** | **R2** | **p-value** |
| --- | --- | --- | --- | --- |
| NS *vs* Rin | 7 | 7.52 | 0.43 | <0.01 |
| NS *vs* WS | 7 | 12.64 | 0.56 | 0.01 |
| NS *vs* WS+Rin | 7 | 13.28 | 0.57 | <0.01 |
| NS *vs* Aph | 7 | 16.01 | 0.62 | <0.01 |
| NS *vs* Rin+Aph | 7 | 5.73 | 0.36 | <0.01 |
| NS *vs* WS+Aph | 7 | 23.54 | 0.7 | <0.01 |
| NS *vs* WS+Rin+Aph | 7 | 3.25 | 0.25 | 0.03 |
| Rin *vs* WS | 7 | 5.95 | 0.37 | 0.02 |
| Rin *vs* WS+Rin | 7 | 5.84 | 0.37 | <0.01 |
| Rin *vs* Aph | 7 | 4.16 | 0.29 | 0.01 |
| Rin *vs* Rin+Aph | 7 | 1.07 | 0.1 | 0.36 |
| Rin *vs* WS+Aph | 7 | 8.8 | 0.47 | <0.01 |
| Rin *vs* WS+Rin+Aph | 7 | 1.92 | 0.16 | 0.11 |
| WS *vs* WS+Rin | 7 | 0.54 | 0.05 | 0.63 |
| WS *vs* Aph | 7 | 2.05 | 0.17 | 0.15 |
| WS *vs* Rin+Aph | 7 | 3.52 | 0.26 | 0.06 |
| WS *vs* WS+Aph | 7 | 2.18 | 0.18 | 0.07 |
| WS *vs* WS+Rin+Aph | 7 | 2.99 | 0.23 | 0.06 |
| WS+Rin *vs* Aph | 7 | 1.96 | 0.16 | 0.17 |
| WS+Rin *vs* Rin+Aph | 7 | 3.22 | 0.24 | 0.04 |
| WS+Rin *vs* WS+Aph | 7 | 0.86 | 0.08 | 0.42 |
| WS+Rin *vs* WS+Rin+Aph | 7 | 3.36 | 0.25 | 0.04 |
| Aph *vs* Rin+Aph | 7 | 1.19 | 0.11 | 0.3 |
| Aph *vs* WS+Aph | 7 | 2.79 | 0.22 | 0.04 |
| Aph *vs* WS+Rin+Aph | 7 | 1.84 | 0.16 | 0.16 |
| Rin+Aph *vs* WS+Aph | 7 | 3 | 0.23 | 0.05 |
| Rin+Aph *vs* WS+Rin+Aph | 7 | 1.3 | 0.11 | 0.28 |
| WS+Aph *vs* WS+Rin+Aph | 7 | 4.26 | 0.3 | 0.02 |
